# Precision Antibody Therapy in Gastric and Gastroesophageal Cancer: Targeting FGFR2b, CLDN18.2, and VEGFR2

**DOI:** 10.3390/cells14211672

**Published:** 2025-10-26

**Authors:** Vivian Chetachi Eziefula Njoku, Yein Lee, Joytish Ramesh, Peter Kubatka, Dietrich Büsselberg

**Affiliations:** 1 Weill Cornell Medicine—Qatar, Doha P.O. Box 24144, Qatar; vce4002@qatar-med.cornell.edu (V.C.E.N.); yel4001@qatar-med.cornell.edu (Y.L.); jor4029@qatar-med.cornell.edu (J.R.); 2 Centre of Experimental and Clinical Regenerative Medicine, Small Animal Clinic, University of Veterinary Medicine and Pharmacy, 041 81 Kosice, Slovakia; peter.kubatka@uvlf.sk

**Keywords:** gastric cancer, gastroesophageal junction cancer, monoclonal antibodies, targeted therapy, bemarituzumab, zolbetuximab, ramucirumab

## Abstract

**Highlights:**

- Precision-targeted antibodies are transforming treatment for GI cancers.
- Bemarituzumab (FGFR2b), Zolbetuximab (CLDN18.2), and Ramucirumab (VEGFR2) demonstrate clinically meaningful survival benefits in phase II–III trials.
- Biomarker-driven patient selection enhances efficacy but poses challenges related to resistance and access.
- Integration of precision antibody therapy requires balancing clinical benefit, cost, and equitable implementation in oncology practice.

**Abstract:**

Gastric and gastroesophageal junction (G/GEJ) adenocarcinomas remain among the most aggressive and lethal malignancies globally. Most patients are diagnosed at advanced stages and respond poorly to conventional chemotherapy, highlighting the urgent demand for more effective, novel treatment strategies such as monoclonal antibody therapies targeting drivers of tumor progression. This review examines the mechanisms, safety profiles, and clinical trial outcomes of three targeted agents—bemarituzumab, zolbetuximab, and ramucirumab—which inhibit tumor growth through the FGFR2b, CLDN18.2, and VEGFR2 pathways, respectively. We also compare traditional versus adaptive clinical trial designs, explore emerging challenges such as therapeutic resistance and treatment-related toxicities, and consider implications for personalized medicine. Collectively, these agents represent a paradigm shift from empiric chemotherapy toward biomarker-driven immunotherapy, with the potential to significantly improve survival and quality of life in patients with advanced G/GEJ cancers.

## 1. Introduction

Gastric and gastroesophageal junction (G/GEJ) adenocarcinomas represent a major global health challenge, ranking as the fifth most diagnosed cancer and third leading cause of cancer-related death worldwide [1]. Incidences are particularly high in East Asia, Eastern Europe, and South America [1,2,3]. In 2020, over 1.1 million new cases were diagnosed globally, with more than 760,000 deaths [1,3]. These findings highlight the aggressiveness of G/GEJ cancers and the need for more effective therapeutic approaches.

Clinicians diagnose most patients with G/GEJ adenocarcinoma at advanced stages, resulting in a poor prognosis and high mortality rate [1,4]. In a previous study, 93.5% of 155 patients had incurable, advanced-stage disease, precluding curative surgery as initial treatment [5,6]. Consequently, treatment typically relies on systemic chemotherapy, which has historically been the first-line standard of care for palliative care [1,7,8]. However, chemotherapy achieves only limited benefits, with low objective response rates (ORRs) and a median overall survival (OS) of roughly 11 months [1,8]. Previous treatments have shown improved survival but due to selective biomarker-defined subset, limited patient population and tumor’s heterogeneity, there is an urgent need for novel, molecularly tailored therapies.

Given these limitations, researchers have increasingly focused on antibody-based approaches that selectively target tumor-associated biomarkers to enhance treatment precision and efficacy. Advances in molecular profiling have identified promising therapeutic targets, including fibroblast growth factor receptor 2b (FGFR2b), claudin 18 isoform 2 (CLDN18.2), and vascular endothelial growth factor receptor 2 (VEGFR2). These targets are clinically relevant due to their selective expression patterns, roles in tumor progression, and association with poor clinical outcomes. Monoclonal antibodies designed against them reflect a shift toward biomarker-guided, personalized treatment strategies in advanced G/GEJ adenocarcinomas.

This review focuses on an evidence-based analysis of three monoclonal antibodies—bemarituzumab, zolbetuximab, and ramucirumab—each targeting distinct molecular pathways involved in tumorigenesis: FGFR2b, CLDN18.2, and VEGFR2, respectively. These agents represent mechanistically distinct but complementary therapeutic strategies that inhibit epithelial growth factor signaling (FGFR2b), disrupt tumor-restricted tight junctions (CLDN18.2), and block pathological angiogenesis (VEGFR2). Developed in response to tumor-specific molecular alterations and advances in biomarker-guided drug design, these therapies enable targeted intervention based on the underlying biology of the disease.

The selection of these agents is therefore driven by their mechanistic diversity, tumor-selective targeting, and alignment with three critical hallmarks of cancer: sustained proliferative signaling (FGFR2b), loss of adhesion and polarity (CLDN18.2), and angiogenesis (VEGFR2). Together, they not only reflect the therapeutic opportunities enabled by molecular profiling but also illustrate how distinct oncogenic pathways can be strategically targeted in a complementary fashion. Collectively, these antibodies exemplify the next generation of biomarker-driven therapies designed to overcome the limitations of conventional chemotherapy and improve outcomes for patients with G/GEJ adenocarcinomas.

This review critically evaluates the mechanistic basis, preclinical findings, and clinical outcomes associated with these antibody-based therapies across Phase I to Phase III trials. By comparing these antibody-based strategies, we aim to evaluate their therapeutic potential, define their advantages and limitations, and define their role within personalized treatment strategies for G/GEJ adenocarcinomas.

## 2. Mechanism

Epithelial tissues predominantly express FGFR2b, a splice isoform (IIIb) of the FGFR2 gene that regulates cell proliferation and differentiation [9]. In G/GEJ adenocarcinomas—particularly the diffuse subtype—aberrant FGFR2b signaling drives tumor growth, lymph node metastasis, and advanced disease progression [10]. FGFR2b resides on the epithelial cell membrane via its extracellular ligand-binding and transmembrane domains [11]. Ligand binding induces receptor dimerization and autophosphorylation, activating downstream pathways that promote tumor cell survival, proliferation, and angiogenesis [12]. FGFR2 gene amplification, found in malignant cells, further amplifies this oncogenic signaling [10]. Meta-analyses indicate that FGFR2 gene amplification occurs in roughly 3–10% of gastric cancers, whereas immunohistochemical assessment demonstrates FGFR2 protein overexpression in a broader subset (~30–40%), implying that protein-level evaluation may identify therapeutically targetable tumors even in the absence of genomic amplification [13].

Immunohistochemical analyses reveal strong FGFR2b membrane staining in tumor tissue [11]. A study of 1974 patients found FGFR2b overexpression (IHC 2+/3+) in 4% of cases, correlating with diffuse histology, nodal metastasis, and poorer survival (H-score ≥ 150; HR 1.84) [10]. In a substantial Japanese cohort analyzed by immunohistochemistry (IHC), FGFR2b overexpression was observed in 88.4%, 42.2%, 22.0%, and 5.8% of cases at scores ≥1, ≥2, ≥3, and ≥4, respectively, indicating widespread but heterogeneous expression across tumors, with the high-expressing subsets representing the most likely candidates for targeted intervention [14]. In contrast, global prescreening for the Phase III FORTITUDE101 trial reported FGFR2b positivity in 37.8% of advanced G/GEJ cases when any 2+/3+ staining was considered, and 16.2% under the stricter threshold (≥2+/3+ in ≥10% of tumor cells) used in bemarituzumab trials [15].

Its epithelial-restricted expression, high membrane accessibility, and well-characterized oncogenic role make FGFR2b an attractive therapeutic target. Bemarituzumab (FPA144), a monoclonal antibody that specifically targets FGFR2b, was developed based on this rationale and has advanced through early- and late-phase clinical trials, including the Phase II FIGHT and pivotal Phase III FORTITUDE-101 studies.

**Figure 1:**
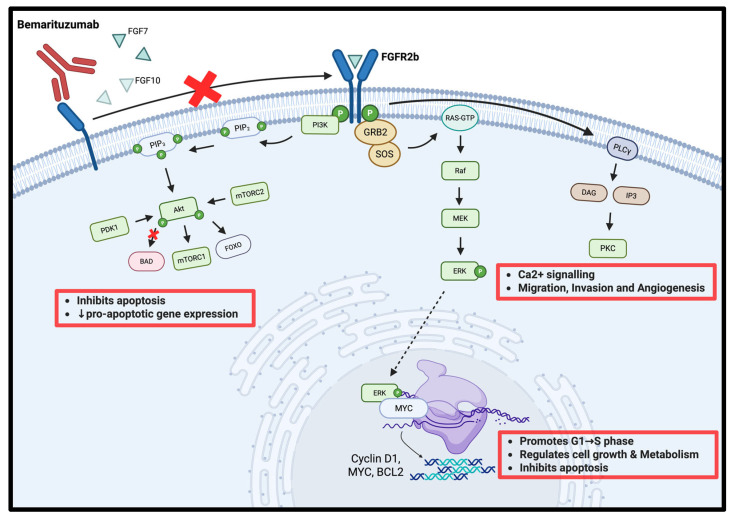
Mechanism of action of bemarituzumab in gastric cancer. The red cross indicates inhibition of FGFR2b dimerization and downstream signaling cascades, thereby suppressing cell proliferation, migration, invasion, and survival. Created with Biorender.com.

Bemarituzumab inhibits FGFR2b activation by blocking the receptor from binding FGF7/FGF10, preventing receptor dimerisation and tyrosine autophosphorylation (Figure 1) [5,16]. Without this blockade, phosphorylated FGFR2b recruits GRB2–SOS, switches RAS-GDP to RAS-GTP, and drives the RAF → MEK → ERK cascade; nuclear ERK with MYC up-regulates Cyclin D1, MYC, and BCL2, pushing G1 → S transition and suppressing apoptosis [17,18]. Parallel phosphotyrosines activate PI3K, generating PIP_3_, which brings in PDK1 and mTORC2 to activate AKT; AKT phosphorylates BAD, inactivates FOXO, and stimulates mTORC1, jointly blocking pro-apoptotic transcription while boosting anabolic metabolism [19,20]. A third branch phosphorylates PLCγ, which cleaves PIP_2_ to IP_3_ and DAG; IP_3_ mobilizes Ca^2+^ and DAG activates PKC, and the resulting Ca^2+^/PKC signals reinforce MAPK and AKT outputs to enhance migration, invasion, and angiogenesis [19,21]. FGFR2b over-expression or amplification—common in gastric cancer—amplifies these pathways and predicts poorer survival [10]. By sealing FGFR2b at the membrane, bemarituzumab simultaneously silences GRB2–SOS–RAS, PI3K–AKT, and PLCγ–PKC signaling, suppressing proliferation, inducing apoptosis, and blunting the invasive, pro-angiogenic phenotype of FGFR2b-addicted tumors [6,16].

The second promising target, CLDN18.2, is a tight junction transmembrane protein selectively expressed in differentiated epithelial cells of the gastric mucosa, where it maintains cell polarity and regulates paracellular permeability [22,23,24]. In healthy gastric tissues, CLDN18.2 remains sequestered within tight junctions, inaccessible to therapeutic agents [25,26]. During malignant transformation, tight junction integrity deteriorates, leading to loss of polarity and relocalization of CLDN18.2 to the extracellular surface of tumor cells [25,27,28]. This altered localization renders CLDN18.2 an accessible and selective therapeutic target. CLDN18.2 was detected in 74.4% of primary gastric cancer specimens (*n* = 367), with roughly 29.4% meeting positivity criteria defined as moderate-to-strong immunohistochemical staining in ≥40% of tumor cells; expression is particularly enriched in diffuse-type and HER2-positive tumors [29].

Because CLDN18.2 is largely absent from non-gastric tissues, therapies targeting this protein carry a low risk of off-target toxicity [22,24,25,26]. Emerging evidence also implicates CLDN18.2 in oncogenic processes such as tumor proliferation and epithelial-to-mesenchymal transition, although these roles require further investigation [23]. Mechanistic investigations indicate that CLDN18.2 facilitates juxtacrine crosstalk between gastric cancer cells and cancer-associated fibroblasts through S100A4, thereby enhancing tumor progression and metastasis in a CAF-dependent fashion [30].

**Figure 2:**
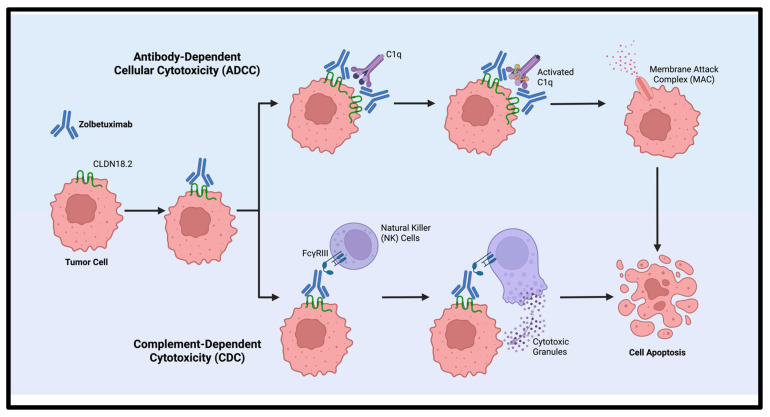
Mechanism of action of zolbetuximab via antibody-dependent cellular cytotoxicity (ADCC) and complement-dependent cytotoxicity (CDC). Created with Biorender.com.

Zolbetuximab, is a first-in-class chimeric IgG1 monoclonal antibody developed to bind CLDN18.2, that exploits this tumor-restricted expression pattern to selectively kill CLDN18.2-positive tumor cells (Figure 2). Upon binding, zolbetuximab mediates antitumor activity through two immune effector mechanisms [31,32,33]:

1. Antibody-dependent cellular cytotoxicity (ADCC): Zolbetuximab binds to CLDN18.2, and its Fc region engages Fc gamma receptor III (FcγRIII)-expressing effector cells, including natural killer (NK) cells and gamma-delta (γδ) T cells [34,35,36]. This interaction activates immune cells to release cytotoxic granules containing perforin and granzymes, leading to pore formation, caspase activation, and apoptosis of tumor cells.
2. Complement-dependent cytotoxicity (CDC): Initiated by Fc-mediated C1q recruitment, which activates the classical complement cascade. This cascade culminates in the assembly of the membrane attack complex (MAC), which disrupts cell membrane integrity, causes osmotic imbalance, and ultimately leads to tumor cell lysis [36].

Angiogenesis is a hallmark of tumor progression, and the vascular endothelial growth factor (VEGF) signaling axis plays a central role. VEGF ligands bind to tyrosine kinase receptors VEGFR1–3, which share similar structures but differ in activation mechanisms, downstream signaling, and biological functions. VEGFR2 (KDR), expressed primarily on endothelial cells, is a high-affinity signaling receptor [37]. It activates pathways that regulate vascular permeability, endothelial proliferation, and migration [37]. Beyond its angiogenic role, VEGFR2 contributes directly to gastric cancer tumorigenesis: overexpression in tumor cells promotes proliferation, migratory capacity, and xenograft tumor growth, in part through upregulation of vitronectin (VTN), a factor associated with adverse clinical outcomes [38]. In gastric cancer, aberrant VEGFR2 signaling drives pathological angiogenesis while simultaneously promoting tumor cell survival, invasion, and immune evasion [39]. Upon binding of VEGF ligands (VEGF-A, -C, -D), VEGFR2 undergoes dimerization and autophosphorylation of its intracellular tyrosine residues. This activation initiates downstream signaling cascades that collectively sustain tumor proliferation, enhance motility and invasive potential, and modulate the tumor immune microenvironment [40,41]. Autocrine VEGF–VEGFR2 signaling in gastric cancer cells stimulates proliferation via a PLCγ–ERK1/2-dependent mechanism, and selective inhibition with apatinib suppresses tumor growth in vitro and in vivo, but predominantly in VEGFR2-high cells, underscoring the functional relevance of autocrine signaling and the potential for biomarker-driven therapy [42].

High VEGFR2 expression occurs in 70–85% of gastric tumor specimens, particularly in advanced disease stages, and correlates with increased metastatic potential and reduced OS [43]. Some studies also report VEGFR2 expression on malignant epithelial cells, suggesting additional autocrine or paracrine oncogenic functions [28]. VEGFR2 upregulation in tumor tissues is a strong prognostic marker, with high expression linked to worse disease-free and overall survival in independent cohorts [38].

**Figure 3:**
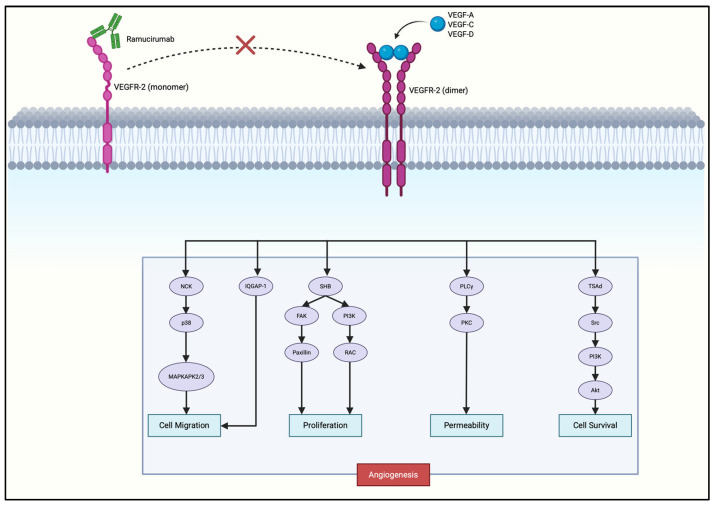
Mechanism of action of ramucirumab in gastric cancer. The red cross indicates inhibition of ligand-induced VEGFR-2 dimerization and downstream signaling, thereby blocking angiogenesis, vascular permeability, and tumor cell survival. Created with Biorender.com.

Ramucirumab, a monoclonal antibody that blocks VEGFR2 signaling, is already FDA-approved for advanced gastric cancer, validating VEGFR2 as a therapeutic target. Ramucirumab binds the extracellular domain of VEGFR2, preventing receptor activation and subsequent downstream pro-angiogenic signaling (Figure 3), thereby effectively inhibiting neovascularization and tumor progression.

Ramucirumab prevents VEGF ligands binding to VEGFR2, preventing receptor dimerization and autophosphorylation at key tyrosine residues (Y951, Y1054, Y1059, Y1175, Y1214), deactivating multiple downstream pathways that promote angiogenesis and tumor progression [39,44,45,46,47]. Blockade of Y1175 activation inhibits PLCγ from hydrolyzing PIP_2_ into DAG and IP_3_. DAG normally activates PKC while IP_3_ increases intracellular Ca^2+^. Both pathways converge on the MAPK/ERK signaling cascade, driving endothelial proliferation [40,48]. Phosphorylated Y951 interacts with adaptor protein TSAd to activate Src kinase, contributing to vascular permeability. Phosphorylated Y1175 recruits SHB, which activates PI3K to convert PIP_2_ into PIP_3_, leading to activation of AKT. AKT activates eNOS, which promotes NO production, while inhibiting pro-apoptotic protein BAD, which both supports endothelial survival and further promotes angiogenesis [41]. SHB also activates FAK, which regulates paxillin, contributing to cell motility and angiogenesis [48]. Y1214 phosphorylation activates the p38/MAPK pathway, which controls cytoskeletal remodeling and cell migration [49].

## 3. Preclinical Evidence

Preclinical research supported the development of targeted monoclonal antibodies—bemarituzumab, zolbetuximab, and ramucirumab—for advanced FGFR2b-, CLDN18.2-, and VEGFR2-positive G/GEJ adenocarcinomas. These agents exhibit direct antitumor effects and immune-modulating properties, supporting their use in future combination strategies.

Researchers developed bemarituzumab after discovering that FGFR2 alterations, like amplification and isoform-specific overexpression, occur in 5–30% of G/GEJ tumors and correlate with poor prognosis and aggressive malignancy. Engineered with an afucosylated Fc domain, bermarituzumab enhances NK cell FcγRIIIa binding, eliciting ADCC. In vitro studies, FGFR2b-overexpressing gastric cancer cell lines demonstrated sub-nanomolar binding affinity, selective growth inhibition, and robust ADCC. Moreover, these studies demonstrated that the antibody exhibited over 20-fold greater affinity for FcγRIIIa banding than its fucosylated counterpart and mediates concentration-dependent suppression of FGFR2 phosphorylation and cell proliferation in SNU-16 gastric cancer cells [16]. In xenograft models, bemarituzumab’s tumor suppression is significantly amplified with chemotherapy—5fluorouracil and oxaliplatin (FOLFOX6)—likely due to simultaneous FGFR2b signaling blockade and immune activation. Mechanistic studies revealed that treatment upregulates PD-L1 expression on tumor cells. Preclinical co-administration with anti–PD-1 antibodies further enhanced tumor regression and durable immune control, establishing bemarituzumab as a dual-acting therapeutic with strong potential for chemo-immunotherapy combinations [16]. Toxicology assessments determined that the antibody is well tolerated at high, non-severely toxic doses in rats (1 mg/kg) and cynomolgus monkeys (100 mg/kg), with pharmacokinetic profiles in monkeys reliably forecasting human Phase 1 pharmacokinetics.

Parallel investigations validated zolbetuximab. In vitro, chemotherapy pretreatment (e.g., with epirubicin, FOLFOX6) increased CLDN18.2 expression theoretically through epigenetic modifications and activation of protein kinase C (PKC) and ERK/MAPK signaling pathways, enhancing zolbetuximab-mediated ADCC and CDC. In vivo xenograft and syngeneic models confirmed that zolbetuximab combined with chemotherapy reduced tumor growth by 93%, compared to 42% for chemotherapy and 65% for zolbetuximab. This combination increased CD8+ T cell infiltration, likely due to tumor lysis and neoantigen release. Combination treatment with anti-PD-1 antibodies further improved tumor suppression, with 50% of treated mice achieving complete tumor regression [26]. 

Complementing these approaches is ramucirumab, an antibody initially developed to inhibit tumor angiogenesis. Preclinical studies showed that VEGFR2 is expressed not only on endothelial cells but also on gastric tumor cells, where autocrine VEGF signaling promotes proliferation and migration. In vitro, ramucirumab inhibited VEGFR2 activation and downstream PLCγ–ERK phosphorylation, leading to reduced tumor cell proliferation and motility [37]. In vivo xenograft models, ramucirumab decreased microvessel density and tumor growth, likely due to tumor vascularization through VEGFR2 blockade [45,50]. Combination therapy with paclitaxel or 5-fluorouracil produced additive or synergistic tumor suppression, while dual blockade of VEGF-A and VEGFR2 overcame adaptive resistance and caused greater tumor suppression [50]. Furthermore, ramucirumab also modulated the tumor microenvironment by reducing immunosuppressive regulatory T cells and altering angiogenesis-related immune factors, supporting its integration into immunotherapy-based regimens [51].

Collectively, these preclinical findings demonstrate that bemarituzumab, zolbetuximab, and ramucirumab exert multifaceted antitumor effects by directly inhibiting oncogenic signaling, enhancing immune-mediated tumor clearance, and reshaping the tumor microenvironment. Comprehensive safety and pharmacokinetic data for bemarituzumab demonstrated immune-mediated synergy across all three antibodies, and strategies to overcome adaptive resistance, particularly through ramucirumab and dual VEGF-A/VEGFR2 inhibition, collectively support their continued clinical development, especially in combination regimens aimed at optimizing durable responses in advanced G/GEJ adenocarcinomas.

## 4. Clinical Development and Trial Results

### 4.1. Phase I Studies

Extensive preclinical studies established the oncogenic roles of FGFR2b, CLDN18.2, and VEGFR2 in gastric cancer, providing a strong rationale for developing targeted monoclonal antibodies—bemarituzumab, zolbetuximab, and ramucirumab. These guided early phase clinical trials to evaluate safety, dosing strategies and assess preliminary efficacy, with a focus on biomarker-based patient selection.

For bemarituzumab, preclinical data showing FGFR2b-driven tumor growth prompted its first-in-human Phase I study (NCT02318329), which enrolled patients with advanced solid tumors and a biomarker-enriched G/GEJ cohort. As summarized in Table 1, this trial identified the recommended Phase II dose, confirmed safety, and demonstrated clinical responses in patients with high FGFR2b expression [52]. An ongoing Asian Phase I/II trial (NCT05322577) is evaluating bemarituzumab in combination with CAPOX or SOX, with or without nivolumab, and preliminary findings reported in Table 1 supporting further combination development [53].

Similarly, zolbetuximab entered clinical evaluation following preclinical evidence of synergy with chemotherapy. Early Phase I trials (NCT00909025 and NCT01671774) are summarized in Table 1 [54,55]. These studies established zolbetuximab’s safety profile, dosing range, and explored immunomodulatory strategies combining zolbetuximab with zoledronic acid (ZA) and interleukin-2 (IL-2) to enhance ADCC [34,36,56,57], providing mechanistic insights for future trials.

Preclinical data supporting VEGFR2 signaling in tumor angiogenesis led to ramucirumab’s early clinical development. As detailed in Table 1, Phase Ib (NCT02359058) and Phase I/II (NCT03008278) studies tested ramucirumab in combination with chemotherapy or the PARP inhibitor olaparib. These studies demonstrated manageable safety, antitumor activity, and the feasibility of chemotherapy-free regimens [58,59].

Collectively, these early-phase studies established Phase II dosing, confirmed tolerability across monotherapy and combination settings, and highlighted the clinical importance of biomarker-driven patient selection. These findings laid the groundwork for ongoing Phase II/III trials and the integration of these antibodies into treatment strategies for advanced G/GEJ adenocarcinomas.

**Table 1:** Summary of Phase I Clinical Trials Evaluating Bemarituzumab, Ramucirumab, and Zolbetuximab in Advanced Gastrointestinal Cancers. This table summarizes Phase I clinical trials conducted to establish the preliminary safety, tolerability, pharmacokinetics, and recommended Phase II dose (RP2D) of bemarituzumab, ramucirumab, and zolbetuximab in patients with advanced gastrointestinal malignancies. Each trial includes dose-escalation and/or dose-expansion cohorts, evaluating monotherapy and combination regimens. Endpoints include incidence of treatment-related adverse events (TRAEs), dose-limiting toxicities (DLTs), maximum tolerated dose (MTD), and early signals of antitumor activity in biomarker-enriched patient subgroups.

Monoclonal Antibody	Trial (NCT #)	Patient Population	Design and Treatment	Dose(s)	Safety	Adverse Events (AEs)	Median Progression-Free Survival (PFS)	Median Overall Survival (OS)	Disease Control Rate (DCR)/Objective Response Rate (ORR)	Key Findings
Bemarituzumab	NCT02318329 [4]	Advanced solid tumors (dose escalation) FGFR2b^+^ G/GEJ adenocarcinoma (expansion); HER2–; late-line	First-in-human, open-label Phase I. Dose escalation → expansion in FGFR2b-high/med/low GEA and FGFR2b^+^ bladder cancer.	0.3–15 mg/kg IV Q2W	No DLTs. Most AEs mild/moderate.	Grade 2 reversible corneal AEs in ~11% at ≥10 mg/kg. Infrequent Grade 3 AEs (~1–2.5%): nausea, neutropenia, anemia, AST/ALP↑, vomiting, infusion reactions. No Grade ≥4 TRAEs	NA	NA	ORR (FGFR2b-high): 17.9% (5/28 PRs) DCR (FGFR2b-high): 64.3% No responses in FGFR2b-low/negative tumors	Confirmed single-agent activity in late-line FGFR2b^+^ GEA. Proof-of-concept established. Well tolerated. Supports further development in combo regimens. Recommended dosing of 15 mg/kg Q2W
	Phase 1/2 Combo Study (NCT05322577) [60]	FGFR2b^+^, HER2– advanced G/GEJ adenocarcinoma; 1st-line, East Asian patients	Ongoing Phase 1/2 in Japan/Asia. Part 1 (safety): Bemarituzumab + CAPOX or SOX ± nivolumab Part 2 (efficacy): Bemarituzumab + SOX + nivolumab (selected regimen)	Bemarituzumab 15 mg/kg Q2W (all arms) CAPOX/SOX per local standards Nivolumab: 240 mg Q2W or 360 mg Q3W	No DLTs reported yet (Part 1 ongoing).	Expected AEs: FGFR2b-related ocular effects, chemo AEs, and immune-related AEs (nivolumab arm)	NA	NA	NA	Aims to identify optimal chemo/immunotherapy partner regimen for bemarituzumab in East Asia. Results will guide global development. Data pending.
Zolbetuximab	NCT00909025 [36,56,61]	15 patients with advanced G/GEJ adenocarcinoma ≥1 prior therapy	First-in-human, open-label, single-dose escalation (5 IV dose cohorts)	Single IV infusion: 33–1000 mg/m^2^	Treatment was well tolerated; No DLTs or discontinuations.	Common AEs: mild-moderate GI effects (nausea, vomiting)	NA	NA	Response: 1 patient with SD (~2 months) at 600 mg/m^2^ dose, no ORR	Linear PK (t_1_/_2_ ~13–24 days), no anti-drug antibodies. 300–600 mg/m^2^ Q2W recommended for Phase II
	NCT01671774 (PILOT) [36,56,61]	28 CLDN18.2^+^ patients with advanced G/GEJ adenocarcinoma ≥1 prior therapy, ECOG ≤1	Open-label with 4 arms: Arm 1: Zolbetuximab + ZA Arm 2: Zolbetuximab + ZA + low-dose IL-2 Arm 3: Zolbetuximab + ZA + mid-dose IL-2 Arm 4: Zolbetuximab monotherapy	Zolbetuximab: 800 mg/m^2^ LD → 600 mg/m^2^ IV Q3W ZA: 4 mg IV (Arms 1–3) IL-2 SC: 1 × 10^6^ IU (Arm 2), 3 × 10^6^ IU (Arm 3)	Treatment was well tolerated.	Most common AEs: Nausea (50%), Vomiting (46%), Fatigue (25%).	Overall 12.7 wks; Arm 4 (mono): 37.3 wks	Overall 40 wks; Arm 3: 60.9 wks	DCR: 55% (11/20); SD: 58%, PD: 42%, no CR/PR	Manageable safety across regimens. Confirmed baseline ADCC. No anti-drug antibodies observed.
Ramucirumab	NCT02359058 [58]	18 Japanese patients with advanced G/GEJ adenocarcinoma; chemo-naïve.	Phase 1b, open-label, multicenter; ramucirumab + one of three chemo regimens: XP (capecitabine + cisplatin) SP (S-1 + cisplatin) SOX (S-1 + oxaliplatin)	Ramucirumab 8 mg/kg on Days 1 and 8 of 3-week cycle + chemo	1 DLT (Grade 3 enterocolitis, SOX).	Common Grade ≥3 AEs: neutropenia (XP: 17%, SP: 50%, SOX: 33%), hypertension (XP: 33%) Other AEs: nausea, constipation, anorexia, HFS	7.6 months (95% CI: 6.0–NE)	NA	ORR: 45.5% DCR: 100%	All regimens showed manageable safety and strong antitumor activity. High serum levels of ramucirumab achieved. Results support further study in randomized trials.
	NCT03008278 [62]	Stage IV G/GEJ adenocarcinoma post ≥1 line of systemic therapy	Phase 1/2, open-label, single-arm; ramucirumab + olaparib (PARP inhibitor)	Ramucirumab + olaparib. Ramucirumab 8 mg/kg Q2W Olaparib twice daily (200/300 mg) Standard 3 + 3 dose-escalation design	1 DLT (Grade 3 fatigue). 3 discontinuations due to toxicity	94% experienced treatment-related AEs: Fatigue (63%) Nausea (59%) Hypertension, vomiting (31% each) Grade ≥3 AEs: 29%	2.8 months (95% CI: 2.3–4.2)	7.3 months (95% CI: 5.7–13.0)	ORR: 14% (6/43) DCR: 40% (16 wks), 17% (24 wks)	Modest activity overall; improved outcomes in homologous recombination deficiency positive (HRD^+^) tumors. ORR and OS numerically higher than ramucirumab monotherapy benchmarks (ORR 3–8%, OS ~5.2 mo). Supports future biomarker-guided trials.

### 4.2. Phase II Studies

Phase II studies of bemarituzumab, zolbetuximab, and ramucirumab advanced biomarker-driven targeted therapy in G/GEJ adenocarcinomas. As summarized in Table 2, these trials evaluated monotherapy and combination regimens, establishing safety profiles, identifying predictive biomarkers and informing Phase III trial design.

For bemarituzumab, the FIGHT trial demonstrated the greatest benefit in patients with high FGFR2b expression establishing the ≥10% cutoff used in Phase III. Safety findings, particularly ocular toxicities, informed treatment discontinuation criteria and monitoring protocols [63]. The ongoing RAINBIRD study represents the first investigation of FGFR2b-targeted therapy in the second-line setting, assessing whether combination with paclitaxel and ramucirumab can improve outcomes beyond current standards [15]. 

Zolbetuximab’s Phase II development included the MONO, FAST, and ILUSTRO trials, which defined its therapeutic potential. Collectively, these trials showed that high CLDN18.2 expression predicts response, combination with chemotherapy improves PFS and OS, and biomarker expressions remain stable over time, supporting its reliability for patient selection [4,23,54,64,65].

Zolbetuximab was assessed in the MONO, FAST, and ILUSTRO trials, which collectively showed that high CLDN18.2 expression predicts response, combination with chemotherapy improves progression-free and overall survival, and biomarker expression remains stable over time, supporting its reliability for patient selection.

Ramucirumab Phase II investigations explored combinations beyond established paclitaxel regimens. The trial evaluating ramucirumab with FOLFIRI demonstrated improved response rates and disease control with manageable safety, although first-line survival benefits were not statistically significant [35].

Collectively, these Phase II studies highlighted the importance of biomarker enrichment, confirmed manageable safety profiles, and laid the foundation for ongoing Phase III trials aimed at refining personalized treatment strategies in advanced G/GEJ adenocarcinoma.

**Table 2:** Summary of Phase II Clinical Trials Evaluating Bemarituzumab, Ramucirumab, and Zolbetuximab in Advanced Gastrointestinal Cancers. This table details Phase II trials designed to assess the efficacy and safety of bemarituzumab, ramucirumab, and zolbetuximab in patients with advanced gastrointestinal cancers, selected based on FGFR2b, VEGFR2, or CLDN18.2 expression, respectively. The trials investigate monotherapy and combination regimens with chemotherapy, including randomized and single-arm designs. Reported outcomes include objective response rate (ORR), disease control rate (DCR), median progression-free survival (PFS), median overall survival (OS), and the incidence of adverse events. These intermediate-phase studies provided critical data that informed the design and progression to Phase III trials.

Monoclonal Antibody	Trial (NCT #)	Patient Population	Design and Treatment	Dose(s)	Safety	Adverse Events (AEs)	Median Progression-Free Survival (PFS)	Median Overall Survival (OS)	Disease Control Rate (DCR)/Objective Response Rates (ORR)	Key Findings
Bemarituzumab	FIGHT (NCT03694522) [6]	FGFR2b^+^ (IHC 2+/3+), HER2–, unresectable/metastatic G/GEJ adenocarcinoma; 1st-line	Global, randomized, double-blind Phase 2 (1:1) Bemarituzumab + mFOLFOX6 vs. Placebo + mFOLFOX6	Bemarituzumab 15 mg/kg Q2W + 7.5 mg/kg Day 8 (Cycle 1) + mFOLFOX6	Higher discontinuation with Bema (40.8% vs. 5.2%) due to ocular AEs	Grade ≥3 TEAEs: 82.9% (Bema) vs. 75.3% (Placebo) Ocular AEs ~67% (Gr 3 27.6%) vs. 10%	9.5 vs. 7.4 months (HR 0.72); not statistically significant overall	19.2 vs. 13.5 months (HR 0.77); trend favoring Bema In FGFR2b ≥ 10% subgroup:	PFS: 14.0 vs. 7.3 months (HR 0.43) OS: 24.7 vs. 11.1 months (HR 0.52)	FGFR2b ≥ 10% subgroup showed marked benefit: ORR 56.5% vs. 36.5%, 2-yr OS 51.3% vs. 21.3%. Justified the ≥10% cutoff for Phase III. Ocular AEs (67%, Gr 3: 27.6%) were the key safety issue.
	RAINBIRD (WJOG 18524G) (Japan, no NCT #) [66]	FGFR2b^+^, HER2– G/GEJ cancer refractory/intolerant to 1 L fluoropyrimidine-platinum ECOG 0–1, measurable disease	Open-label, single-arm Phase II (Japan) Bemarituzumab + Ramucirumab + Paclitaxel (2nd-line treatment)	Bema 15 mg/kg Q2W + Ramucirumab 8 mg/kg Q2W + Paclitaxel 80 mg/m^2^ (Days 1, 8, 15, xsssssz28-day cycle)	Ongoing	AEs expected: FGFR2b-related ocular toxicity (prespecified monitoring), VEGFR2- and taxane-class toxicity. No results yet.	Ongoing—no efficacy data reported yet	Ongoing—no efficacy data reported yet	Ongoing—no efficacy data reported yet	First study to test Bema beyond 1 L. Designed to assess whether Bema can improve outcomes when added to standard 2 L regimen. May extend FGFR2b-targeting benefit to later-line settings. Results pending.
Zolbetuximab	NCT01197885 (MONO) [33,56,61]	54 patients with recurrent/refractory CLDN18.2^+^ (≥50%) G/GEJ adenocarcinoma; ECOG 0–1.	Phase IIa, open-label, multicenter 3-cohort monotherapy (dose escalation + expansion).	Cohort 1: 300 mg/m^2^ Q2W Cohorts 2 and 3: 600 mg/m^2^ IV Q2W cy	Well tolerated; no DLTs or discontinuations.	Common Grade 1–2 AEs: nausea (61%), vomiting (50%), fatigue (22%)	NA	NA	ORR: 9% (4 PRs; all ≥70% CLDN18.2) DCR: 23% (4 PR + 6 SD)	Activity limited to high CLDN18.2 expressers. No anti-drug antibodies. Supports biomarker-driven selection.
	NCT01630083 (FAST) [25,56,61]	252 patients with advanced/metastatic CLDN18.2^+^ G/GEJ cancer; ECOG 0–1.	Randomized (1:1:1) Phase II trial: Arm 1: EOX Arm 2: Zolbetuximab + EOX Arm 3 (exploratory): Zolbetuximab + EOX Maintenance: Zolbetuximab monotherapy.	Arm 2: 800 mg/m^2^ (loading, Cycle 1 Day 1) → 600 mg/m^2^ Q3W Arm 3: 1000 mg/m^2^ Q3W EOX administered every 3 weeks	Manageable profile.	AEs in ZOL vs. EOX alone: Nausea (75% vs. 52%), vomiting (66% vs. 31%) Grade ≥3 AEs: neutropenia (23% vs. 14%), nausea (8% vs. 4%), vomiting (8% vs. 1%)	Arm 2 vs. 1: 7.5 vs. 5.3 months (HR 0.44, *p* < 0.0005) ≥70% CLDN18.2: 9.0 vs. 5.7 months (HR 0.38)	Arm 2 vs. 1: 13.0 vs. 8.3 months (HR 0.55, *p* < 0.0005) ≥70% CLDN18.2: 16.5 vs. 8.9 months (HR 0.50)	ORR: 39% (ZOL + EOX) vs. 25% (EOX) DCR: 83% vs. 76%	Significant efficacy in combination arm, especially in ≥70% CLDN18.2 expressers. Favorable risk-benefit profile supports Phase III development.
	NCT03505320 (ILUSTRO) [54]	54 HER2–, CLDN18.2^+^ (≥75%) advanced/metastatic G/GEJ adenocarcinoma.	Open-label multicohort trial: 1A: ZOL monotherapy (≥3rd-line) 2: ZOL + mFOLFOX6 (1st-line) 3A: ZOL + pembrolizumab (≥3rd-line)	NA	Manageable safety across cohorts.	Common AEs: nausea (up to 90.5%), vomiting, anemia, neutropenia. Grade ≥3 (Cohort 2): neutropenia (28.6%), anemia (9.5%), and pain (9.5%)	1A: 1.54 months 2: 17.8 months 3A: 2.96 months	1A: 5.62 months 2, 3A: Not yet reached	ORR: 0% (1A), 71.4% (2), 0% (3A) DCR: 44–55.6% (1A), 100% (2), 66.7% (3A)	ZOL + mFOLFOX6 in 1st-line showed high efficacy. Monotherapy and IO combo (3A) less effective in late-line. CLDN18.2 expression remained stable over time (61.1% concordance archival vs. fresh biopsy).
Ramucirumab	NCT03081143 [35]	111 patients with advanced G/GEJ adenocarcinoma previously treated with platinum/fluoropyrimidine	Phase II, randomized, open-label: FOLFIRI + ramucirumab vs. FOLFIRI alone	Ramucirumab 8 mg/kg IV Q2W + FOLFIRI (irinotecan 180, leucovorin 400, 5-FU bolus 400 + inf. 2400 mg/m^2^/46 h) Q2W	Well tolerated; consistent with known profiles	Grade ≥3 neutropenia, diarrhea, hypertension in combo arm	HR: 0.73 (27% risk reduction) HR: 0.49 in prior-docetaxel pts	6-months OS rate: 54% in combo arm HR: 0.97 (similar OS)	ORR: 22% vs. 11% (combo vs. FOLFIRI alone)	Ramucirumab + FOLFIRI improved PFS and doubled ORR, especially in docetaxel-pretreated patients. Feasible and tolerable as second-line option.
	NCT01246960 [67]	168 untreated patients with advanced G/GEJ adenocarcinoma	Phase II, randomized, double-blind, placebo-controlled: ramucirumab + mFOLFOX6 vs. placebo + mFOLFOX6.	Ramucirumab 8 mg/kg Q2W + mFOLFOX6 (oxaliplatin 85, leucovorin 400, 5-FU bolus 400 + inf. 2400 mg/m^2^/46 h) Q2W.	Safety profile manageable and comparable between arms	Common Grade ≥3 AEs: neutropenia, hypertension, thrombocytopenia	5.6 months (ramu) vs. 6.0 months (placebo); not significant	11.7 months (ramu) vs. 11.5 months (placebo); not significant	DCR >50% in both arms; ORR not significantly different	Combo was safe but did not improve PFS or OS. Preliminary activity supported further investigation of ramucirumab in first-line setting.

### 4.3. Phase III Studies

The clinical development of targeted monoclonal antibodies for advanced G/GEJ adenocarcinoma has significantly progressed, with Phase III trials evaluating bemarituzumab, zolbetuximab, and ramucirumab. These studies highlight biomarker-guided therapy as a standard of care in first- and second-line treatment settings, with details of the trial’s characteristics and outcomes summarized in Table 3.

Bemarituzumab, targeting FGFR2b, demonstrated statistically significant OS benefit in the FORTITUDE101 Phase III trial, establishing the first success for FGFR2b-directed. Safety findings were consistent with prior studies, with ocular toxicities being the most notable but generally manageable [52]. The ongoing FORTITUDE102 study is assessing bemarituzumab in combination with nivolumab and chemotherapy to explore potential synergistic effects in biomarker-selected patients, with results expected in 2025 [68].

Zolbetuximab, targeting CLDN18.2, has also shown survival benefits in first-line treatment. The SPOTLIGHT trial combined zolbetuximab with mFOLFOX6 [22,55,56,61], whereas the GLOW trial used CAPOX as the chemotherapy backbone [23,31,69]. Despite differences regimen and geographic distribution, both studies demonstrated significant improvements in PFS and OS compared to chemotherapy alone [21,23,69,70,71]. SPOTLIGHT reported more pronounced disease control and longer survival [64], while GLOW confirmed efficacy across treatment backbones and patient subgroups, reinforcing the reproducibility of zolbetuximab’s benefit [69]. Gastrointestinal adverse effects, primarily nausea and vomiting, were common but clinically manageable [22,71].

Ramucirumab represents the first antiangiogenic therapy to show a survival advantage in this disease. In second-line settings, the REGARD and RAINBOW Phase III trials demonstrated improved OS with ramucirumab, either as monotherapy or combined with paclitaxel, compared to control regimens. Toxicities were manageable and consistent with known profiles. However, predictive biomarkers for ramucirumab response remain unidentified, highlighting the need for improved patient selection [64,72].

Together, these Phase III studies provide evidence supporting the integration of monoclonal antibody therapies into advanced G/GEJ adenocarcinoma management. Bemarituzumab and zolbetuximab have advanced personalized first line, while ramucirumab remains an effective second-line antiangiogenic therapy.

**Table 3:** Summary of Phase III Clinical Trials Evaluating Bemarituzumab, Ramucirumab, and Zolbetuximab in Patients with Advanced Biomarker-Positive Gastrointestinal Cancers. This table presents an integrated overview of Phase III clinical trials assessing three monoclonal antibodies—bemarituzumab (targeting FGFR2b), ramucirumab (targeting VEGFR2), and zolbetuximab (targeting CLDN18.2)—in patients with advanced gastrointestinal (GI) cancers. Trials are grouped according to the biomarker expression status (FGFR2b+, VEGFR2+, and CLDN18.2+) and provide detailed information on study design, treatment arms, dosing regimens, safety profiles, progression-free survival (PFS), overall survival (OS), and response metrics such as objective response rate (ORR) and disease control rate (DCR). These studies aim to evaluate the efficacy and tolerability of precision-targeted therapies in biomarker-selected GI cancer populations, including G/GEJ adenocarcinomas.

Monoclonal Antibody	Trial (NCT #)	Patient Population	Design and Treatment	Dose(s)	Safety	Adverse Events (AEs)	Median Progression-Free Survival (PFS)	Median Overall Survival (OS)	Disease Control Rate (DCR)/Objective Response Rates (ORR)	Key Findings
Bemarituzumab	FORTITUDE-101 Phase III (NCT05052801) [15]	FGFR2b-overexpressing, HER2-negative unresectable locally advanced/metastatic G/GEJ adenocarcinoma; no prior advanced therapy	Global, randomized, double-blind Phase III (547 pts, ~300 sites) Bemarituzumab + mFOLFOX6 vs. placebo + mFOLFOX6 (1:1)	Bemarituzumab 15 mg/kg IV Q2W (with initial Day 8 dose) + mFOLFOX6 (standard dosing)	Overall tolerability consistent with Phase 2 No new/unexpected toxicities	Common AEs (>25% with bemarituzumab): Ocular disturbances, (higher frequency/severity vs. placebo) Chemotherapy related: anemia, neutropenia, nausea Other: fatigue, peripheral neuropathy, stomatitis (similar incidence in both arms)	Pending full data	met primary endpoint (significantly improved vs. placebo)—exact median not released yet	Pending full data	First Phase 3 success for FGFR2b-targeted therapy in this setting. Bemarituzumab + chemo significantly improves OS in FGFR2b+, HER2– G/GEJ cancer. Ocular side effects are manageable. Full efficacy data awaited.
	FORTITUDE-102 Phase III (NCT05111626) [68]	FGFR2b-positive, HER2-negative advanced G/GEJ adenocarcinoma, first-line setting	Phase 1b/3 trial: Part 1: open-label safety run-in of bemarituzumab + mFOLFOX6 + nivolumab Part 2: randomized double-blind Phase 3 comparing triplet vs. placebo + chemo + nivolumab	Bemarituzumab 15 mg/kg Q2W + mFOLFOX6 + nivolumab (standard dose)	Ongoing trial; no unexpected safety issues flagged so far Ocular toxicity monitored closely with addition of nivolumab	Expected AEs similar to FORTITUDE-101 plus potential immune-related effects due to nivolumab Detailed AE data not yet available	NA (ongoing)	NA (ongoing)	NA (ongoing)	No efficacy data reported yet (ongoing) Evaluates benefit of adding bemarituzumab to chemo + anti-PD1 immunotherapy in FGFR2b+ patients. Could establish new triplet regimen if positive. Results anticipated 2025.
Zolbetuximab	NCT03504397 (SPOTLIGHT) [33]	565 untreated, CLDN18.2^+^ (≥75%), HER2^−^ G/GEJ adenocarcinoma ECOG 0–1	Phase III, randomized, double-blind, placebo-controlled Zolbetuximab + mFOLFOX6 vs. Placebo + mFOLFOX6	Zolbetuximab: 800 mg/m^2^ LD → 600 mg/m^2^ Q3W mFOLFOX6: biweekly	≥Grade 3 TEAEs: 87% (Z) vs. 78% (P) Serious AEs: ~45% both arms Treatment-related deaths: 5 (Z) vs. 4 (P) Discontinuation due to AEs: 9% (Z) vs. 4% (P)	Nausea: 81% vs. 61% (≥G3: 8.7% vs. 2.4%) Vomiting: 64.5% vs. 34.5% (≥G3: 12.2% vs. 3.6%) Decreased appetite: 47% vs. 33.5% Other common AEs: anemia, neutropenia, stomatitis, fatigue, neuropathy Grade ≥3 anemia/neutropenia: similar between groups	10.61 vs. 8.67 months	18.23 vs. 15.54 months	ORR: 60.7% vs. 62.1% DCR: Not reported	Zolbetuximab + mFOLFOX6 significantly prolonged PFS and OS despite similar ORR. Benefit driven by durable disease control. Supports use of zolbetuximab as 1L therapy in CLDN18.2^+^ G/GEJ cancers.
	NCT03653507 (GLOW) [69,73]	507 untreated, CLDN18.2^+^ (≥75%), HER2^−^ G/GEJ adenocarcinoma ECOG 0–1	Phase III, randomized, double-blind, placebo-controlled Zolbetuximab + CAPOX vs. Placebo + CAPOX	Zolbetuximab: 800 mg/m^2^ LD → 600 mg/m^2^ Q3W CAPOX: Q3W for 8 cycles	≥Grade 3 TEAEs: 72.8% (Z) vs. 69.9% (P) Discontinuation due to AEs: 7.1% (Z) vs. 4.4% (P) No new safety signals; toxicity manageable	Vomiting: 12.2% vs. 3.6% (≥G3) Nausea: 8.7% vs. 2.4% (≥G3) Anemia: 10.6% vs. 11.2% Neutropenia: 10.2% vs. 9.6%	8.21 vs. 6.80 months (HR = 0.687; *p* = 0.0007) 12-months PFS rate: 29% vs. 17%	14.39 vs. 12.16 months (HR = 0.771; *p* = 0.0118)	ORR: 42.5% vs. 40.3% DCR: 3.1%/50.8% vs. 1.5%/47.3%	Zolbetuximab + CAPOX significantly prolonged PFS and OS. Benefits driven by disease stabilization rather than tumor shrinkage. Confirms CLDN18.2 as a relevant target in 1L G/GEJ cancer.
Ramucirumab	NCT00917384 [64]	355 patients with advanced G/GEJ cancer, post-first-line platinum/fluoropyrimidine	Phase III, randomized, double-blind, placebo-controlled (Ramucirumab vs. placebo)	Ramucirumab 8 mg/kg IV Q2W	Well tolerated overall	Grade ≥3 AEs: Hypertension (8%), Abdominal pain (6%), Asthenia (6%), Anemia (6%)	2.1 vs. 1.3 mo (HR 0.48; *p* < 0.0001)	5.2 vs. 3.8 mo (HR 0.77; *p* = 0.047)	DCR: 28% vs. 16% ORR: Low	Ramucirumab significantly improved PFS and OS. While ORR was low, disease stabilization was clinically meaningful. First anti-VEGFR agent to show survival benefit in gastric cancer.
	NCT01170663 [72]	665 patients with advanced G/GEJ adenocarcinoma progressing after first-line therapy	Phase III, randomized, double-blind (Ramucirumab + paclitaxel vs. placebo + paclitaxel)	Ramucirumab 8 mg/kg IV (Days 1 and 15) + Paclitaxel 80 mg/m^2^ (Days 1, 8, 15) of 28-day cycle	Toxicities were manageable and consistent with known profiles	Grade ≥3 AEs: Neutropenia (41% vs. 19%), Hypertension (14% vs. 2%), Leukopenia, Fatigue, Anemia	4.4 vs. 2.9 mo (HR 0.64; *p* < 0.0001)	9.6 vs. 7.4 mo (HR 0.81; *p* = 0.017)	ORR: 28% vs. 16% DCR: 80% vs. 64%	Ramucirumab + paclitaxel significantly improved survival and became the standard second-line regimen. Benefits were consistent across subgroups, including patients with poor ECOG status and across regions.

## 5. Discussion

### 5.1. Limitations and Challenges

#### 5.1.1. Biomarker Complexity and Patient Selection

The biggest barrier with the implementation of antibody-based strategies in G/GEJ cancer is diagnostic ambiguity. FGFR2b expression is heterogeneous both within and between tumors. In the pivotal FIGHT study, roughly one in six screened specimens tested FGFR2b-positive by IHC, yet only a minority showed amplification [5,10,15]. This complicates assay strategy: protein-based cut-off (≥10% 2+/3+ cells) are simple but risks enrolling tumors driven by alternate pathway, whereas gene-centric approach (FISH or next-generation sequencing) may miss bona fide protein-overexpressing tumors lacking copy-number gain.

Similar dilemmas surround CLDN18.2, whose staining patterns vary between primary and metastatic sites. Subgroup analyses of SPOTLIGHT and GLOW showed maximal benefit from zolbetuximab in tumors with homogenous, strong staining in ≥75% of cells, while lower-expression cohorts showed reduced hazard ratio separation [55,69]. Thus, any given assay threshold simultaneously governs therapeutic reach and specificity. CLDN18.2 expression demonstrates substantial intratumoral and intersite variability. In a metastatic gastric cancer cohort (*n* = 166), approximately 38% of surgical specimens exhibited intratumoral heterogeneity, while ~25% showed discordance between primary and metastatic lesions. Peritoneal metastases displayed the highest CLDN18.2 positivity (44.3%) and concordance (31.4%), highlighting sampling bias risks [74]

Ramucirumab, which targets endothelial VEGFR2 rather than tumor-intrinsic proteins, circumvents heterogeneity but suffers from the absence of a validated predictive biomarker, limiting enrichment for highly responsive patients [60,61]. Exploratory analyses have linked circulating angiopoietin-2 (Ang-2) as a potential predictive biomarker, suggesting a prognostic role; however, its predative value for ramucirumab response remains unproven [75].

#### 5.1.2. Therapeutic Resistance

Even in biomarker-selected populations, adaptive resistance can limit response durability. For bemarituzumab, up-regulation of alternative receptor-tyrosine-kinase pathways—including MET, HER2, and EGFR—has been documented in pre-clinical CRISPR and phospho-proteomic screens, providing a plausible bypass route [45]. A kinome-wide CRISPR/Cas9 screen in FGFR2-amplified gastric cancer cell lines (e.g., KatoIII) demonstrated that loss of focal adhesion kinase (ILK) and suppression of EGFR/HER2 signaling increased sensitivity to FGFR inhibition. Moreover, combined blockade of these pathways enhanced FGFR inhibitor efficacy, identifying EGFR/HER2 as key escape mechanisms under FGFR2 blockade [76]. Immunohistochemistry also suggests that sub-clonal FGFR2b loss under selective pressure can effectively de-target the antibody. Zolbetuximab faces different challenges: as its mechanism relies on Fc-mediated ADCC and complement activation, tumors with impaired immune-synapse formation or heightened complement regulation may attenuate cytotoxicity [26,33]. In several MONO- and FAST-derived biopsies, CLDN18.2 remained detectable at progression, implying that downstream immune evasion rather than target loss was operative [25,33]. Ramucirumab resistance is often framed in terms of “angiogenic escape,” wherein VEGF-independent pro-angiogenic circuits—most notably FGF2 signaling [77]—along with ANGPTL4 and related mediators, re-establish vascular supply despite ongoing VEGFR2 blockade [50,51]. A clinical biomarker analysis revealed that, in patients receiving ramucirumab plus paclitaxel, elevations in circulating VEGF-C and angiopoietin-2 levels at disease progression correlated with poorer clinical outcomes, implicating these ligands as potential mediators or indicators of adaptive angiogenic escape [51]. Multiomic data from serial biopsies corroborate this concept, revealing dynamic induction of FGF ligands, Tie2 signaling, and VEGF-C/D family members after initial response. 

#### 5.1.3. Safety Considerations

With adequate monitoring, all three antibodies exhibit acceptable tolerability profiles, yet each carries a distinctive toxicity signature. FGFR2b inhibition perturbs corneal epithelial homeostasis, producing dry eye, punctate keratitis, and occasional ulceration that may reduce dose intensity if unmanaged. In the Phase I bemarituzumab trial, corneal adverse events, including dry eye (17.7%), keratitis (15.8%), and punctate keratitis (14.5%), occurred in nearly two-thirds of patients receiving bemarituzumab + mFOLFOX6, with some progressing to limbal stem cell deficiency [6]. The bemarituzumab program, therefore, implemented a dedicated ophthalmic grading scale integrating corneal fluorescein staining and best-corrected visual acuity [62]. Preclinical rodent studies demonstrated corneal epithelial thinning and meibomian gland atrophy, highlighting the importance of vigilant ocular monitoring in clinical settings [78].

Zolbetuximab’s main safety liabilities—nausea, vomiting, and infusion reactions—are thought to reflect on-target binding to gastric mucosa and mast-cell degranulation; anti-emetic prophylaxis plus slowed infusion counters most events [56,58,59]. In ferret models, zolbetuximab induced rapid onset vomiting due to mucosal irritation, which was mitigated by pre-treatment with fosaprepitant and dexamethasone [79].

Ramucirumab carries the class-effect toxicities of VEGF-pathway inhibition. Hypertension is manageable with ACE inhibitors or calcium-channel blockers, whereas proteinuria, thrombo-embolism, and bleeding demand closer scrutiny, especially in combination regimens incorporating anticoagulants [64,72]. A meta-analysis of global Phase III trials identified bleeding, hypertension, edema, and proteinuria as the most frequent grade ≥3 adverse events associated with ramucirumab across indications. In hypertensive gastric cancer patients receiving ramucirumab, concomitant treatment with renin–angiotensin system inhibitors (RAS-I) were associated with a significantly lower incidence of proteinuria compared with calcium channel blockers, supporting preferential RAS-I use in this context [80].

#### 5.1.4. Economic and Access Barriers

High acquisition costs threaten to limit equitable uptake. Contemporary cost-effectiveness studies estimate incremental cost-effectiveness ratios (ICERs) for zolbetuximab plus chemotherapy above accepted willingness-to-pay (WTP) thresholds: A Chinese cost-effectiveness analysis reported an incremental cost-effectiveness ratio (ICER) of approximately $388,186 per quality-adjusted life year (QALY) gained for zolbetuximab plus CAPOX compared with placebo plus CAPOX, far exceeding China’s WTP threshold (~$38,223/QALY). A separate comparative model for the United States and China estimated ICERs of ~$821,516/QALY and ~$273,568/QALY, respectively, indicating that zolbetuximab is not cost-effective in either market without substantial price reductions [73].

Ramucirumab negotiated conditional reimbursement in several countries only after real-world post-authorization safety studies and price-volume agreements. In mainland China, a cost-effectiveness analysis indicated that ramucirumab plus paclitaxel would only meet cost-effectiveness criteria if ramucirumab’s price dropped below approximately $244 per 4-week cycle, corresponding to an ICER of $26,014/QALY. In Japan, comparisons of ramucirumab plus paclitaxel (Ram + PTX) versus PTX or CPT-11 alone produced cost-effectiveness ratios of JPY 2,780,432.4 per month of survival gain (≈$20,000) and JPY 2,185,179.0 per month (≈$15,000), respectively, both markedly higher than for chemotherapy alone, highlighting persistent concerns over economic viability [81].

Bemarituzumab’s eventual ICER will hinge on the magnitude of the overall-survival improvement in FORTITUDE-101 and the breadth of FGFR2b-positive prevalence; payer appetite is likely to demand biomarker-defined deployment to cap budget impact. Health-economic modeling will be essential to assess whether the survival benefits of bemarituzumab justify reimbursement, especially if its use is restricted to patients with high FGFR2b expression.

## 6. Conclusions

Gastric and gastroesophageal junction adenocarcinomas remain a significant health issue due to late-stage diagnoses, pronounced tumor heterogeneity, and limited efficacy of standard chemotherapy. These challenges highlight the need for biologically informed treatment strategies.

Monoclonal antibody therapies—bemarituzumab (FGFR2b), zolbetuximab (CLDN18.2), and ramucirumab (VEGFR2)—represent a precision oncology approach targeting key cancer mechanisms: oncogenic signaling, epithelial antigen-driven proliferation, and angiogenesis. Ramucirumab has shown antiangiogenic efficacy across multiple tumor types, while bemarituzumab and zolbetuximab are advancing through late-phase trials as a personalized treatment option for biomarker-selected patients.

However, successful integration depends on standardized biomarker tests, which are limited by variability in assay availability, cost, and standardization across clinical settings. Overcoming these barriers is essential to ensure patient selection and equitable access.

Ultimately, these therapies signal a shift toward individualized, biomarker-driven treatment in gastric cancer, with the potential to improve outcomes through continued innovation, rigorous clinical validation, and practical implementation.

## Data Availability

No new data were created or analyzed in this study. Data sharing is not applicable to this article.

## Abbreviations

The following abbreviations are used in this manuscript:

ADCC	antibody-dependent cellular cytotoxicity
AE	adverse event
AKT	protein kinase B
CAPOX	capecitabine + oxaliplatin
CDC	complement-dependent cytotoxicity
CI	confidence interval
CLDN18.2	claudin-18 isoform 2
CPS	combined positive score (PD-L1 scoring method)
CR	complete response
DAG	diacylglycerol
DCR	disease control rate
DLT	dose-limiting toxicity
ECOG	Eastern Cooperative Oncology Group performance status
ERK	extracellular signal-regulated kinase
FGFR2b	fibroblast growth factor receptor 2b isoform (IIIb)
FOLFIRI	folinic acid + 5-fluorouracil + irinotecan
FOLFOX/mFOLFOX6	(modified) folinic acid + 5-fluorouracil + oxaliplatin
G/GEJ	gastric/gastroesophageal junction
HER2	human epidermal growth factor receptor 2
HR	hazard ratio
HRD	homologous recombination deficiency
IHC	immunohistochemistry
IL-2	interleukin-2
IP_3_	inositol-1,4,5-trisphosphate
IV	intravenous
LD	loading dose
MAC	membrane attack complex
MAPK	mitogen-activated protein kinase
mAb	monoclonal antibody
NA	not applicable
NK cell	natural killer cell
ORR	objective response rate
OS	overall survival
PD-1	programmed cell death protein-1
PD-L1	programmed death-ligand 1
PFS	progression-free survival
PI3K	phosphoinositide 3-kinase
PIP_2_	phosphatidylinositol-4,5-bisphosphate
PIP_3_	phosphatidylinositol-3,4,5-trisphosphate
PKC	protein kinase C
PLCγ	phospholipase C-gamma
PR	partial response
Q2W/Q3W	every 2 weeks/every 3 weeks
RP2D	recommended Phase 2 dose
SAE	serious adverse event
SOX	S-1 (tegafur/gimeracil/oteracil) + oxaliplatin
SP	S-1 + cisplatin
TEAE	treatment-emergent adverse event
TRAE	treatment-related adverse event
VEGF	vascular endothelial growth factor
VEGFR2 (KDR)	vascular endothelial growth factor receptor-2
XP	capecitabine + cisplatin
ZA	zoledronic acid
ZOL	zolbetuximab
